# Paraplegia Caused by a Large Spinal Epidural Hematoma 12 Days After Thoracic Decompression and Fusion

**DOI:** 10.7759/cureus.75140

**Published:** 2024-12-05

**Authors:** Kaoru Morooka, Akihiko Inokuchi, Teiyu Izumi, Ryuta Imamura, Takeshi Arizono

**Affiliations:** 1 Orthopaedic Surgery, Kyushu Central Hospital of the Mutual Aid Association of Public School Teachers, Fukuoka, JPN

**Keywords:** delayed onset hematoma, emergency hematoma evacuation, lower limb paralysis, neurological deficits, ossification of posterior longitudinal ligament, postoperative spinal epidural hematoma (seh), risk factors for seh, spinal stenosis surgery, thoracic spine surgery

## Abstract

A 41-year-old man with a history of obesity, hypertension, and smoking suffered from numbness and weakness in both lower limbs. He was diagnosed with ossification of the posterior longitudinal ligament and ligamentum flavum in the cervical and thoracic spine by X-rays, CT, and MRI. The patient underwent laminectomies at T2 and T3 levels, along with posterior fusion from T1 to T4, to address an upper thoracic spine lesion causing sensory deficits up to T5 and gait disturbances. The surgeries were T2 and T3 laminectomies and posterior T1-T4 fusion. The intraoperative and postoperative courses were uneventful. However, on the twelfth postoperative day, the patient suddenly experienced severe back pain followed by complete paralysis of both lower extremities, accompanied by significant swelling at the surgical site. An urgent MRI revealed a large hematoma dorsal to the dura mater, extending from T1 to T4. Emergent evacuation of the hematoma was performed, and no active bleeding was observed. Over time, a gradual improvement in the strength of the lower limbs was observed. Follow-up at two years postoperatively indicated the patient could walk unassisted for up to 1 km, although he continued to experience nocturnal urinary incontinence and erectile dysfunction. This case highlights the importance of recognizing that postoperative spinal epidural hematoma can develop even more than 10 days after spinal surgery. Patient and staff education is crucial to ensure prompt recognition and intervention.

## Introduction

Postoperative spinal epidural hematoma (SEH) is a rare yet severe complication that can lead to significant neurological deficits. The incidence of postoperative symptomatic SEH is estimated at approximately 0.52%, with delayed onset occurring in about 0.16% of patients who underwent spinal surgery, typically more than 72 hours after surgery [1]. Delayed SEH, developing more than a week postoperatively, is particularly uncommon and challenging to diagnose due to its insidious onset without a clear precipitating event [2,3]. We report a rare case of delayed postoperative SEH occurring 12 days after laminectomy and posterior fusion in a patient with ossification of the posterior longitudinal ligament and ligamentum flavum, resulting in sudden, complete paralysis. This case underscores the importance of considering SEH even in the late postoperative period and highlights the need for prompt intervention to prevent permanent neurological damage.

## Case presentation

A 41-year-old obese man presented with numbness and weakness in his both lower limbs. He had hypertension and a history of smoking. His symptoms started with numbness in the right lower limb, which gradually worsened over several months. As the symptoms progressed, he experienced numbness in the opposite leg and pain in both legs, leading to unstable walking. He reported intermittent claudication after walking 100 meters, along with a feeling of incomplete bladder emptying and a weak urinary stream.

On physical examination, his height was 175.3 cm and weight was 129.4 kg, resulting in a body mass index (BMI) of 42.1. Manual muscle testing showed mild weakness in both quadriceps (grade 5−) while other lower limb muscle groups exhibited normal strength. There were no signs of hyperreflexia, hyporeflexia, or pathological reflexes. Lumbar extension was restricted. Straight leg raise and femoral nerve stretch tests were negative. Sensory examination revealed numbness and loss of vibration sense in both feet. Jackson and Spurling's tests were negative. Grip strength on a hand dynamometer was 46 kg on the right and 40 kg on the left.

**Figure 1:**
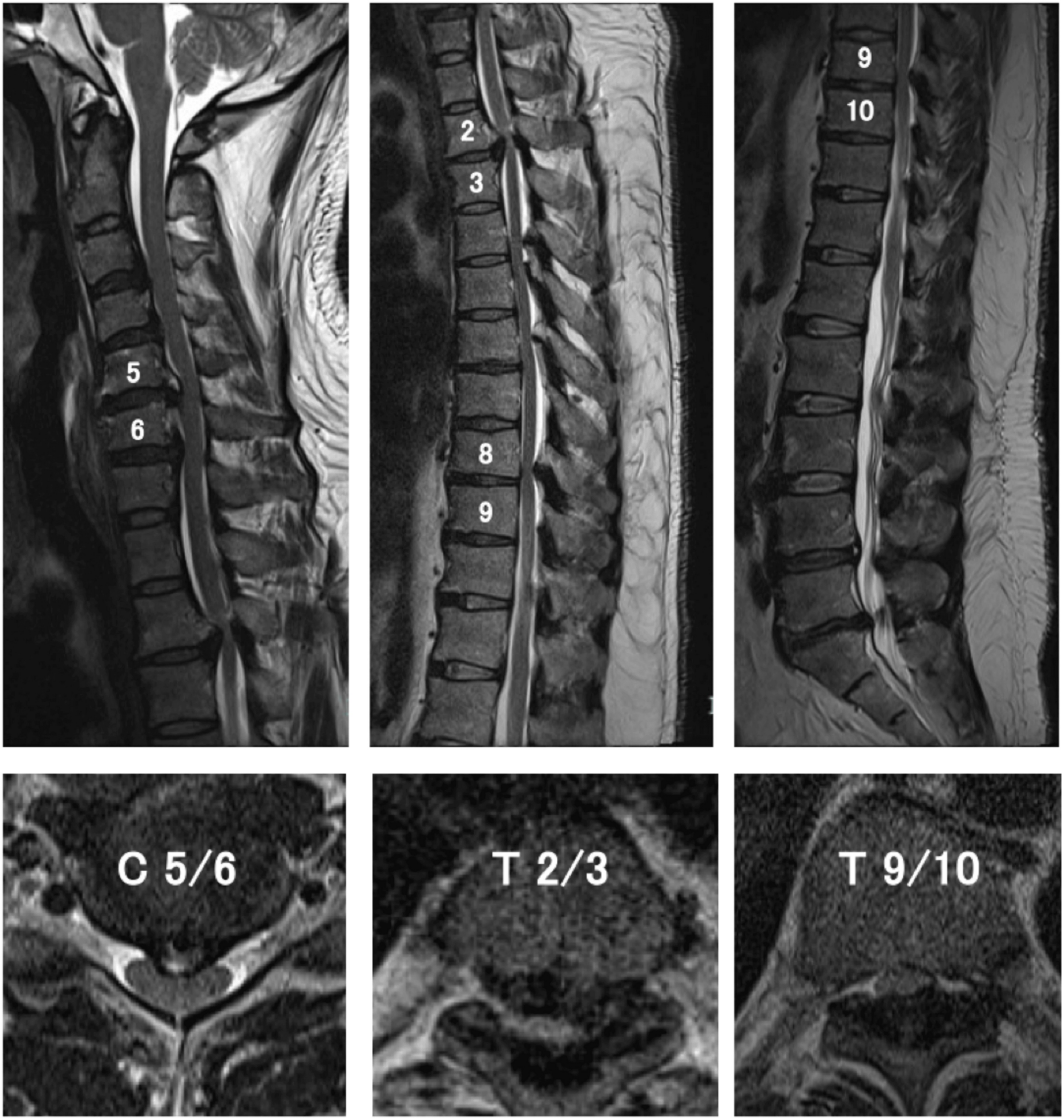
Sagittal and axial T2-weighted imaging showed spinal canal stenosis caused by ossification of the posterior longitudinal ligament and ligamentum flavum extending from the C3/4 to the C6/7 interspaces and at the T2/3 and T9/10 levels. Spinal cord compression and signal change in the spinal cord are visualized at T2/3.

**Figure 2:**
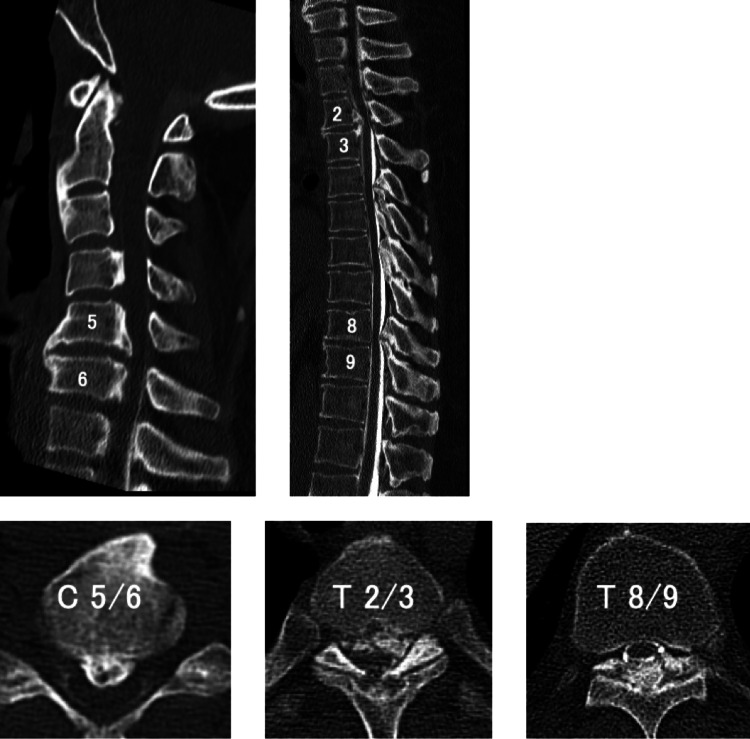
Computed tomography myelography of the spine showed ossification of the posterior longitudinal ligament from C3 to C7 and at T2/3. Ossification of the ligamentum flavum was noted from T4 to T10.

Magnetic resonance imaging (MRI) and computed tomography myelography of the spine showed spinal stenosis due to ossification of the posterior longitudinal ligament and ligamentum flavum extending from the C3/4 to the C6/7 interspaces and at the T2/3 and T9/10 levels. Flattening of the spinal cord with a change in signal intensity was noted at the T2/3 level (Figures 1, 2 ). He was diagnosed with ossification of the posterior longitudinal ligament and ligamentum flavum in the cervical and thoracic spine was diagnosed. There were multiple intervertebral compressions, and although the level of disability could not be determined from the neurological findings, we determined that the more superior lesions, rather than lumbar lesions, were responsible for his disabilities. After consulting with the patient, we decided to first perform surgery on the upper thoracic spine, where the compression was the strongest and the brightness changes in the spinal cord on MRI were also strong.

The patient underwent T2 and T3 laminectomies with posterior T1-T4 fusion. Despite the absence of epidural fat, there was no adhesion between the dura mater and the ossified ligamentum flavum. Two drains were placed in the epidural space before wound closure. Because of the patient's severe obesity, the surgical field was deep, and intraoperative fluoroscopic visualization was challenging. As a result, the operation time was longer than usual (3 hours and 54 minutes). The estimated blood loss volume was 587 mL.

**Figure 3:**
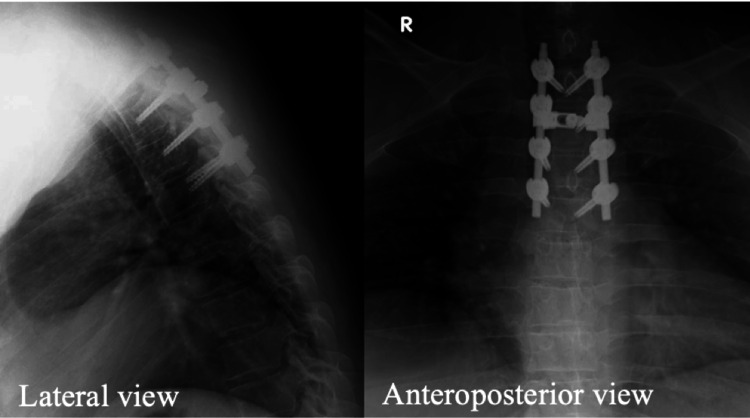
Anteroposterior and lateral plain radiography one week after surgery

Upon awakening from anesthesia, the patient exhibited no issues with lower limb movement. Mobilization began on the first postoperative day. The drain fluid was initially bloody and output decreased to 10 mL/hour by the second day after surgery, when the drains were removed. The total drainage volume over the first 2 days was 580 mL. Postoperative radiography showed good placement of the thoracic pedicle screws and rods (Figure 3).

**Figure 4:**
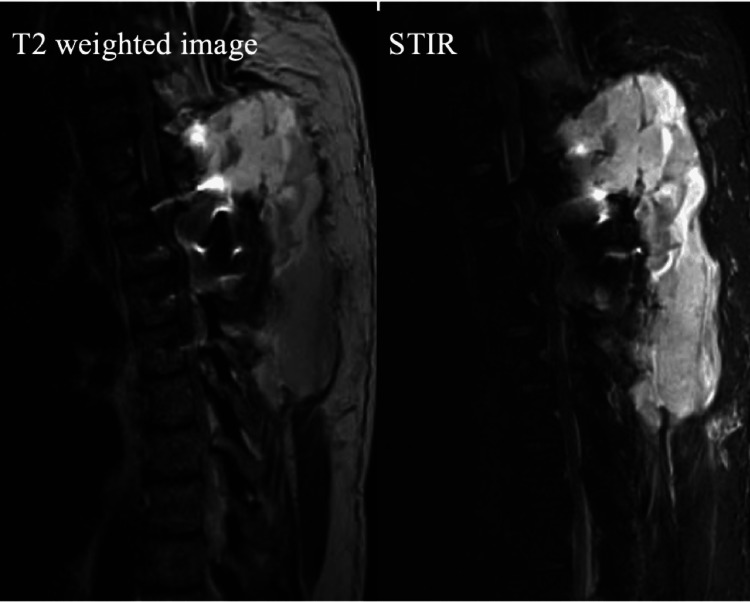
Emergency magnetic resonance imaging of the thoracic spine on postoperative day 12. Extensive fluid accumulation is visualized dorsal to the dura from C7 to T5.

**Figure 5:**
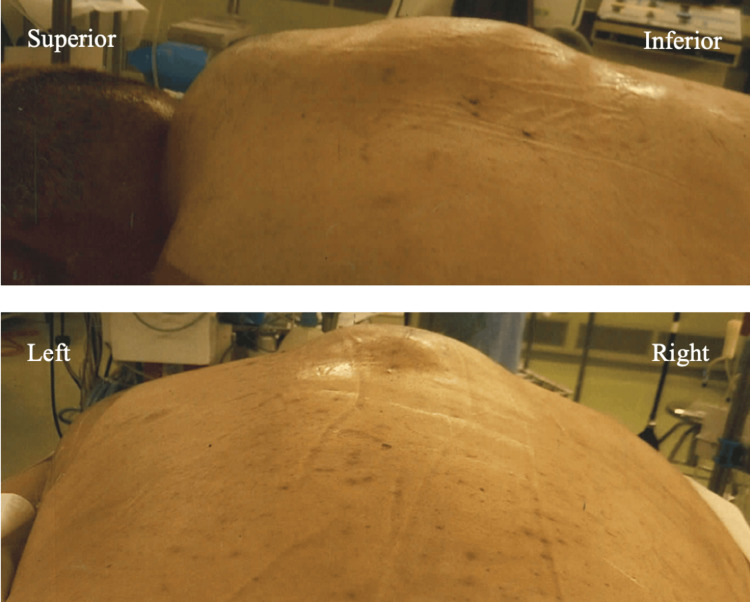
Photographs of the patient in the operating room show considerable swelling at the surgical site before emergency hematoma evacuation surgery was performed.

**Figure 6:**
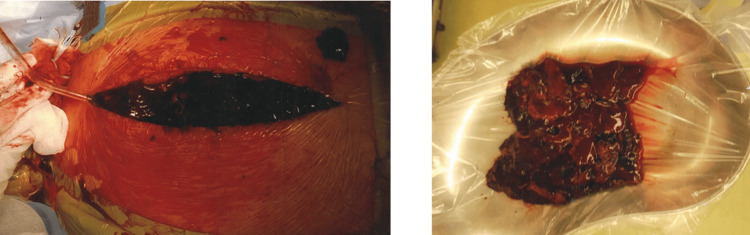
Intraoperative photography before and after hematoma removal shows large, well-organized blood clots.

On the twelfth day after surgery, the patient began experiencing back pain, which rapidly worsened over two hours. He subsequently developed paraplegia (grade 0 muscle strength bilaterally), and significant swelling was observed at the surgical site. Aspiration of the surgical site yielded approximately 50 mL of blood. The patient’s blood pressure was stable at 120/80 mmHg. Blood testing earlier that day showed a white blood cell count of 12,200/µL, a neutrophil ratio of 59.5%, and a C-reactive protein level of 1.79 mg/dL. Emergency MRI revealed a large hematoma dorsal to the dura mater from T1 to T4 (Figure 4). Only sagittal images could be obtained because of the patient’s pain. Emergency hematoma evacuation surgery was then performed (Figures 5, 6). Although a large blood clot was identified, no active source of bleeding was found. Operation time was 63 minutes with an intraoperative blood loss of 450 mL. Immediately after surgery, the patient’s lower limb muscle strength remained 0 with a loss of all lower limb sensations except for touch. On preoperative and immediately postoperative coagulation tests, the only abnormality was a slightly prolonged prothrombin time after surgery (14.3 seconds; reference range, 9.5-12.0 seconds); prothrombin time activity, international normalized ratio, and activated partial thromboplastin time were all within normal limits. Two months after the hematoma evacuation, a gradual improvement in lower limb muscle strength was observed. At the four-month follow-up, he was able to walk with parallel bars and was transferred to a rehabilitation facility. Over time, his walking ability continued to improve. Two years after the hematoma evacuation, the patient was able to walk 1 km without assistance; however, he experienced persistent nocturnal urinary incontinence and erectile dysfunction.

## Discussion

Previous reports have identified various risk factors associated with postoperative SEH, including advanced age, obesity, a history of hypertension, alcohol consumption, smoking, blood coagulation disorders, multilevel surgery, reoperation, intraoperative use of gelatin sponges, blockage of the surgical drainage tube, abnormal drainage, anticoagulant use, incomplete hemostasis, surgery lasting longer than 120 minutes, and blood loss exceeding 600 mL [2,4-11]. In a systematic review and meta-analysis, Qian et al. suggested that spinal deformity, spinal tumors, and minimally invasive surgery may be associated with an increased incidence of postoperative SEH [1]. Conversely, idiopathic SEH has been associated with minor trauma, chiropractic therapy, Paget’s disease of the spine, ankylosing spondylitis, and rheumatoid arthritis [12-15].

The overall incidence of symptomatic postoperative SEH in the aforementioned meta-analysis was 0.52%, with a 0.16% incidence of delayed postoperative SEH (onset >72 hours after surgery) [1]. In a retrospective study of 3371 spinal surgeries, Anno et al. reported a 0.42% incidence of symptomatic SEH, with almost half occurring during the delayed phase (a mean onset was 5.0 ± 1.1 days after surgery) [3]. Wang et al. reported a mean onset time of 130.60 ± 61.78 hours (range, 3-14 days) in the study focused on delayed SEH after lumbar surgery [2]. Hao et al. and Hu et al. have both reported cases of lumbar SEH occurring as late as postoperative day 14 [16,17].

Our patient developed a large acute thoracic hematoma 12 days after surgery, which is relatively late compared to typical cases; notably, there was no apparent trigger. The hematoma developed rapidly and resulted in severe neurological deficits. Although potential risk factors, such as multilevel surgery, prolonged operative time, obesity, hypertension, and smoking, may have contributed to the development of SEH, none of these are particularly exceptional. Furthermore, the delayed onset of the hematoma, occurring without any identifiable cause and resulting in complete paralysis within a short time frame, is extremely rare based on our review of the literature.

The involvement of Batson’s epidural venous plexus has been suggested as a cause of postoperative SEH [18]. However, in rapidly progressing cases, rupture of local arteries has also been implicated [19]. In the case reported by Hao et al., postoperative SEH was caused by a rupture of the anterior branch of the fourth lumbar artery [16]. Our patient developed complete paralysis within three hours of onset, suggesting possible involvement of Batson’s plexus or rupture of a local artery. However, no obvious source of bleeding was identified during surgery. Nonetheless, the prompt intervention and rapid hematoma removal likely contributed to the patient’s neurological recovery. It is essential to recognize that acute paralysis caused by a postoperative SEH can occur even after the first postoperative week without any specific trigger. Educating patients and medical staff about this possibility is essential to ensure timely intervention when necessary.

## Conclusions

We encountered a patient who developed a large postoperative spinal epidural hematoma (SEH) on the twelfth postoperative day, resulting in severe neurological impairment within a few hours. Intraoperative findings did not reveal the source of the bleeding. It is essential to recognize that a potentially catastrophic postoperative SEH can occur more than 10 days after surgery, even in the absence of specific risk factors or an evident trigger.
